# The Etiology of Catarrh

**Published:** 1884-12

**Authors:** George F. Burder

**Affiliations:** Consulting Physician Bristol General Hospital President Bristol Medico-Chirurgical Society, October, 1884


THE BRISTOL

Medico-Chirurgical Journal.

DECEMBER, 1884.

### THE ETIOLOGY OF CATARRH.

### Inaugural Address

BY

GEORGE F. BURDER, M.D., F.R.C.P.,

*Consulting Physician Bristol General Hospital*

*President Bristol Medico-Chirurgical Society, October,* 1884.

In no branch of medical science is greater activity to be observed in the present day than in the investigation of the causes of disease, and in none is there larger promise of benefit to mankind. Successful modes of treatment may be suggested by analogy and confirmed by experiment, even where the nature of the disease is but dimly discerned, and its essential cause unknown. But it can scarcely be doubted that a more exact knowledge upon these points will sooner or later lead to important results. Not therapeutics only, but hygiene also, must be greatly helped by the development of sound views on the causation of disease. From the practical point of view, therefore, no less than from the purely scientific, one cannot but regard very hopefully the researches of Koch and others on the part played by microscopic organisms in the production of disease. We may differ among ourselves in our readiness to accept new doctrines (and it is wise not to be hasty); but no one, I think, can fail to see that year by year our knowledge in this direction is being extended and consolidated. Nor is it only by the labours of microscopists that these results are achieved. The volume lately issued by the Collective Investigation Committee of the British Medical Association supplies us with a large body of evidence pointing to the conclusion that acute pneumonia is, in some cases at least, a specific disease, depending therefore, in all probability, like other specific diseases, upon the presence of a microscopic organism.

The subject of my address this evening is not without relation to these inquiries. I propose to ask your attention to certain views which I have long held on the Etiology of Catarrh.

The most widely prevalent idea regarding the origin of catarrh is that which finds expression in the term by which in this country we commonly designate the disorder. But the term "cold" can scarcely be said to cover all the various agencies to which catarrhs are popularly attributed. One individual catches cold, as he believes, from walking in his garden without his hat, although the air to which he is exposed may be anything but cold. Another takes the complaint from sitting in a current of air between a window and a door, although the movement of air in the room may be trifling compared with that which out of doors he would consider healthful. A change from winter to summer clothing is generally considered to be attended with danger. Some people will take cold through reading a newspaper fresh from the press, and I suppose we have all met with nervous patients who have been rendered uneasy about their own health by our entering their apartment with damp clothes.

But leaving the vagaries of hypochondriacs, we find in authors of repute a striking consensus of opinion on the action of cold as the exciting cause of catarrh.

In Cullen's *Practice of Physic* (1786) we read: "The remote cause of catarrh is most commonly cold applied to the body." And again: "The application of cold which occasions a catarrh probably operates by diminishing the perspiration usually made by the skin, and which is therefore determined to the mucous membrane of the air-passages. As a part of the weight which the body daily loses by insensible evacuation is owing to an exhalation from the lungs, there is probably a connection between this exhalation and the cutaneous perspiration, so that the one may be increased in proportion as the other is diminished; and therefore we may understand how the diminution of cutaneous perspiration, in consequence of the application of cold, may increase the afflux of fluids to the lungs, and thereby produce a catarrh." Cullen goes on to speak of the epidemic or (as he calls it) contagious catarrh. "There are," he says, "two species of catarrh. One of these, as I suppose, is produced by cold alone, and the other seems manifestly to be produced by a specific contagion. Of such contagious catarrhs, I have pointed out many instances occurring from the fourteenth century down to the present day. In all these instances the phenomena have been much the same; and the disease has always been particularly remarkable in this, that it has been the most widely and generally spreading epidemic known."

Dr. John Brown, who was a pupil and contemporary of Cullen, and who was given to turning the world upside down with his theories, taught a quite opposite doctrine respecting the origin of catarrh. "Heat alone," he says, "fulness of diet, strong drink, and moderate exercise, for certain produce it; cold, cold water, spare diet, and rest, as certainly and effectually remove it. It was, therefore, a very unlucky mistake to think it arose from cold alone, and was to be cured by heat. The occurrence of catarrh so often in summer, where its action can be a thousand times traced back to heat, but never to cold; the influenza never needing the assistance of cold to induce it, which catarrh often does; its never succeeding to pure cold, but immediately to heat—facts known to old women, to shoemakers and taylors, to blear-eyed beggars and barbers, unknown to medical authors and professors—are all circumstances that confirm the same fact."

But Brown's views left no permanent impression on medical opinion; for, coming down to our own day, I read in Sir Thomas Watson's *Lectures*: "Catarrh is the commonest of all disorders. Not one man in ten thousand passes a winter without having a *cold* of some sort. And this name points to its ordinary cause—cold somehow applied to the body. It does not always or often result, I apprehend, from cold air brought into contact with the membrane itself in the process of breathing; but from cold, and especially from cold and wet, applied to the external integument."

But Watson, like Cullen, recognises that the great epidemics of catarrh cannot owe their origin to cold. "The facts that have now been mentioned," he writes,

"respecting the influenza, warrant, I think, the conclusion that it does *not* depend upon any mutations in the ordinary and obvious qualities of the atmosphere; upon any degrees or variations, I mean, of its temperature, its motions, or its moisture; upon what is expressed in the single word *weather*." Of Schönbein's theory, connecting epidemic catarrh with the presence in the atmosphere of an excessive quantity of ozone, Watson speaks with some favour, regarding it as not unworthy of careful investigation. He continues: "Another hypothesis, more fanciful perhaps, at first sight, than these, yet quite as easily accommodated to the known phenomena of the distemper, attributes it to the presence of innumerable minute substances, endowed with vegetable or with animal life, and developed in unusual abundance under specific states of the atmosphere, in which they float, and by which they are carried hither and thither. Myriads of these animalcules, or of these vegetable germs, coming in contact with the mucous membranes, and especially with that of the air-passages, irritate (it is imagined) those surfaces, and exercise a poisonous influence upon the system. Now the sporules of certain fungi, which ruin the health and destroy the vitality of larger plants, on which they prey, are inconceivably small. I shall prove to you presently that vegetable effluvia are capable of producing, in the human body, symptoms not very dissimilar from those of influenza. Again, that the waters of this globe swarm with living creatures, which are invisible by our unaided eyes, the microscope has taught us. Others, too minute to be estimated even by that wonder-showing instrument, in all probability exist. We cannot doubt that the gaseous fluid which surrounds this planet equally teems with living atoms. We know that multitudes of insects, and of cryptogamous plants, infinite in number with respect to our finite powers of computation, *are* sometimes suddenly hatched or developed in places which were previously free from them. It is easy to conceive that atmospheric infusoria (so to speak) may rapidly congregate, or vivify, in masses sufficient to render deleterious the very air we breathe. If this be so, we can understand how such a cause of disease may first act here and there, and presently overspread large districts; how it may move, or be wafted, from place to place, or be carried about by persons; how its course and operation may be circumscribed and definite; and how some germs or ova may remain after the visit, retaining their vitality, and ready in future seasons again to start into life and activity under favouring circumstances. Taking the insect hypothesis, and knowing, as we do, that some animal poisons (that of small-pox, for example) have the singular property of multiplying themselves in the human body, like yeast in beer, we may conceive that diseases, produced by animalcules, may thus infect the fluids of the body, and become contagious in the fullest sense of that term."

This zymotic theory, you will observe, is only applied by Watson to the great epidemics of catarrh which from time to time sweep over large tracts of country.

Niemeyer, in his *Text-book,* ascribes ordinary catarrh to "chilling of the external skin, and the action upon it of a sudden change of temperature;" but admits that "we cannot satisfactorily account for the phenomenon." He adds that "in very many cases the exciting causes of catarrh are unknown, unless we accept the explanation with which people usually content themselves, that 'they must have taken cold somewhere.'"

Lastly, to bring my references down to the latest date, I find in Quain's *Dictionary* nothing further respecting the causation of catarrh than the bare statement, that it "commences shortly after exposure to cold, or more particularly to cold and damp."

In the face of authorities such as I have quoted, and in the face (as I suppose) of almost universal medical opinion, it may seem presumptuous in me to question the direct connection between cold and catarrh. Nevertheless, such is my contention; and I can only hope that the inequality of the conditions may not lead my hearers to regard with preconceived disfavour the arguments by which I hope to defend my position.

My first argument will be based upon the similarity—I might say the identity—of the symptoms observed in a common cold with those observed in the wide-spreading epidemics of influenza. The difference appears to me to be one of intensity merely—intensity in respect of the geographical area involved, intensity in respect of the number of persons attacked within a given area, and (following necessarily from this) intensity in respect of the absolute number of severe, dangerous, or fatal cases. I need not dwell upon the points of resemblance. It will be sufficient if I remind you, on the one hand, that by common consent we speak of the severer forms of ordinary catarrh as "influenza colds;" while, on the other hand, it is clear from the published records that in the most serious epidemics of influenza a large proportion of cases have not, even in point of severity, differed from the common run of colds. I will go further, and say that what is known as sporadic catarrh, by way of distinguishing it from the widespread epidemics just referred to, is very often itself epidemic in a minor degree. This is a matter of common observation, although the lesson to be learned from it has been (as I think) strangely overlooked.*

*Primâ facie,* then, there is good ground for supposing that influenza-catarrh and common catarrh are one and the same disease, and that any theory of causation which can be proved of the one is equally true of the other. Now it is but little that we know definitely of the exciting cause of influenza; but there is one thing that we know about it with absolute certainty. The recorded history of influenza epidemics positively precludes the supposition that the attacks are due to anything appertaining specially to the individuals attacked. This remark is true of all epidemic diseases, but influenza is an epidemic of epidemics. The observation of Cullen already quoted is as true to-day as it was a hundred years ago. Influenza is beyond doubt "the most widely and generally spreading epidemic known." Of an epidemic in 1557, it is recorded by a contemporary writer that the disease attacked all parts of Spain at once, and that the greatest part of the people of that kingdom were seized with it almost on the same day. Dr. Glass, of Exeter, describing the epidemic of 1775, writes: "On the 11th or 12th of November it made its appearance in the Devon and Exeter Hospital, and within a week seized 173 persons, being all the

* It may be said that the apparently epidemic character of common catarrh admits of being explained by a reference to the weather prevailing at the time when such epidemic character is observed. I do not think this is in any case a satisfactory explanation; but there is a class of cases in which it conspicuously fails. Some years ago I was staying with a party of friends at Torquay. The month was August; the weather fine and summer-like. The party consisted at one time or another of eight persons, and of those eight, five became affected with catarrh. Instances of this kind are not uncommon, and receive no explanation from any of the current theories of catarrh.

servants and patients then in the house, except two children; 162 of them were coughing together." At St. Petersburgh, in 1782, it is related that forty thousand persons were taken ill in one morning with influenza.

Now no one in his senses would suggest in explanation of these phenomena that all these crowds of people in Spain, Exeter, and St. Petersburgh had been walking in the garden without their hats, or sitting in damp boots, or indulging in any of the other indiscretions to which it is customary to attribute sporadic catarrh. Nothing can be more certain than that the exciting cause of influenza (whatever be its precise nature) is to be found in some general influence pervading the entire community; and to my mind it is hardly less clear that common catarrh, essentially identical as it is with influenza, not only in its symptoms, but even in its epidemic tendency, owns a similar origin.

But here crops up a difficulty arising from individual experience. "You may theorise as you please," I hear in imagination some one say, "but experience is better than theory, and you will never persuade me that my cold was not caught last Sunday in church through sitting beneath an open window. I felt the draught, and was certain at the time that I was taking cold."

"Experientia fallax," says the Father of Medicine; and without inquiring too curiously whether Hippocrates meant his words in the sense in which we understand them, I take them as embodying, in that sense, a profoundly wise caution. Experience can only tell the subject of a cold that he is the subject of a cold. It cannot tell him how he contracted it. That is a matter of theory more or less probable, and I think it is not difficult to show that the theory commonly adopted is exceedingly improbable.

For, in the first place, the cause assigned seems altogether inadequate to the effect produced. There is no one engaged in the ordinary affairs of life who does not continually expose himself to far greater and more sudden changes of temperature than would be involved in sitting by an open window. If the latter is sufficient to induce catarrh, what shall we say to the exposure of the naked body in a cold bath? True, the one is intentional, the other accidental and unsought; but is it rational to suppose that the effect will be determined by circumstances such as these?

Again, there is a source of fallacy in the impression which the subjects of catarrh often have regarding the time and circumstances of their taking cold. In many cases one of the early symptoms of catarrh is a sense of chilliness. This is naturally attributed to external causes, and the individual traces his illness to the particular exposure which he suffered at that time. In truth, the sensation was but the mild representative of the rigor with which febrile disorders are commonly ushered in. Precisely the same error is continually made with regard to more serious diseases. A patient with typhoid fever, or even with small-pox, may indicate the precise occasion on which he took the "chill" which he believes induced his disease, ignorant of the fact that for many days previously the disease had been breeding in his system, and was only then beginning to assert its presence. The initial symptom is mistaken for the exciting cause. This fallacy may go a long way to explain the widespread belief that catarrh is due to cold—a belief which I hold to be an error, but an error almost hopeless of eradication, seeing that it has struck its roots into our very language.

Another source of fallacy may be mentioned. Exposure to cold air may directly excite sneezing, and as sneezing is a symptom of catarrh, the person affected concludes that he has taken cold; and if the cold should not develop, he will perhaps flatter himself that he has warded it off by prompt recourse to hot foot-baths and gruel. But sneezing, although a symptom of catarrh, is not catarrh, and in the case supposed has nothing to do with catarrh.

If the circumstances to which catarrhs are commonly attributed were circumstances of an exceptional character, it would be right to attach a certain weight to the popular judgment respecting them. But the case is far otherwise. The assumed causes are for the most part occurrences of the most commonplace kind, continually happening without observed consequences, but now and again followed by a cold. *Post hoc, ergo propter hoc.* The many instances in which a draught or a shower is encountered without harm following either pass unnoticed, or, fitting into no theory, are soon forgotten. The few instances in which a cold follows arrest the attention and become impressed upon the memory. The supposed exciting causes being so common as they are, and the complaint attributed to them being so common too, it would be strange if occasional coincidences did not occur, though no causal relation existed between the two.

Dr. Tanner, in his *Practice of Medicine*, while adopting in general the view of other writers with regard to the origin of catarrh, adds the following qualification: "It arises not from mere cold, but from too sudden a change of atmosphere or from exposure to wet, &c., when the strength is exhausted. . . . The application of cold is only dangerous when the heated body, exhausted by exercise, is rapidly parting with its caloric." Assuming this qualification to be reasonable, I would hang upon it another argument. Of all the persons who are able (as they believe) to refer their colds to a particular exposure, how many, I ask, would be able truthfully to say that at the time of exposure their heated bodies were exhausted by exercise? Surely a very small proportion. But if that be so, it follows that a very large proportion of the colds which are believed to be accounted for are really not accounted for at all; that is to say, the great majority are as yet without any further explanation than that with which, as Niemeyer tells us, people usually content themselves, that "they must have taken cold somewhere." The absence of explanation hitherto must be allowed to be a strong point in favour of an explanation offered.

I have read somewhere a theory of catarrh which has not, I think, found its way into our text-books. According to this view, cold applied to the skin is the exciting cause, but the effect is produced through the agency of the nervous system in a reflex manner. This explanation is plausible, and meets one of the objections that I have raised to the common view—namely, that of disproportion between cause and effect. It is characteristic of reflex nervous phenomena that there is no necessary proportion between the cause and the effect. The touch of a feather may induce a shiver, which the dead weight of a crowbar would fail to excite. But are there (I would ask) any positive observations warranting the idea that the application to the skin of an agent *per se* innocuous is capable of producing in this reflex manner hyperæmia of distant parts? I greatly doubt it. Besides, if the air in which we live and move, and which is never constant in temperature, were capable, with every trifling change, of setting up internal disease, it is difficult to see how life could be maintained at all.

It might help us to some further knowledge of the etiology of catarrh if statistics were available respecting this very common complaint—too common, apparently, to have been thought worthy of statistical treatment. In the absence of any better material, I may be excused if I fall back upon some records of personal experience. I have been in the habit of noting regularly the colds from which I have myself suffered, and I find that my record extends now over twenty-four years. The total number of colds recorded during that interval is 82, giving an average of about 3½ per annum. The number may seem large; but I should observe that it includes colds of all degrees of severity and duration. Thus, out of the 82, 8 were noted as having a duration of two days only, and 12 a duration of three days. The mean duration of the whole number was nine days, the disorder not being considered at an end so long as any trace remained. If the two-day colds were excluded, as not worthy to be considered developed colds, the number would be reduced to 74, or 3 per annum. But for my present purpose the slight colds are as valuable as the more severe, both alike indicating the presence of an exciting cause. No calendar year in my series was without a cold; but there was once an interval of twelve months, from February to February. The annual totals ranged from one to seven. Judged by Sir Thomas Watson's assertion, that not one man in ten thousand passes a winter without a cold, I must have enjoyed more than an average immunity. Twice have I passed unscathed through what may be called the five winter months (November — March), and five times through the shorter winter comprised in the three central months of that season (December—February).

But if my statistics are to be relied upon, the winter is not, in point of fact, the season when catarrhs most prevail. The highest of my monthly totals occurs in April; the lowest in July. April furnished no less than 15 colds in the twenty-four years; July only one. The winter months occupy an intermediate position, the numbers ranging from 6 to 9.*

Considering the length of time over which the observations extend, and the large inequality in the monthly totals, and weighing these two circumstances against the admitted disadvantage attaching to results derived from a single person's experience, I am disposed to think it may be possible to deduce from the figures some conclusions which may not be altogether undeserving of trust.

Why should the month of April be specially prolific of catarrh, far more so than either of the winter months? This fact, at all events, does not point to cold as the essential cause; still less does it point to wet, for April is the driest of all the months.

But it will be safer not to rely upon the evidence of single months. By grouping the months in accordance with their meteorological characters, we may look for more reliable results.

And first we will group them in accordance with their mean temperature,† comparing the three coldest months (December, January, and February) with the three

* The following is a complete list of the monthly totals:—January, 9; February, 9; March, 7; April, 15; May, 7; June, 4; July, 1; August, 2; September, 6; October, 10; November, 6; December, 6.

† The meteorological data quoted in this address are all derived from observations made at Clifton.

warmest months (June, July, and August). In the three coldest months I find 24 catarrhs; in the three warmest, only 7. Here, then, is evidence that atmospheric cold, although it may not be the essential cause, is an important factor in determining the occurrence of catarrh.

To discover, if possible, the influence of rainfall, I first of all compare the three driest months (February, March, and April) with the three wettest months (August, September, and October). In the three driest months, with a mean aggregate rainfall of 6·7 inches, I find 31 catarrhs, the largest number in any three months; in the three wettest months, with a mean aggregate rainfall of 10·7 inches, I find only 18 catarrhs. Here we have a prevalence of catarrh coinciding with comparative drought, and a rarity of catarrh coinciding with abundant rainfall. This comparison, however, is less conclusive than the former, because the effect of temperature, if considered to be established by the first comparison, may be held sufficient to explain the difference in the second comparison; for the three wettest months, although not the warmest, are comparatively warm, and the three driest months, although not the coldest, are comparatively cold.

A more instructive comparison may be instituted between the three coldest months (December, January, and February) and the three driest months (February, March, and April). The mean temperature of the first of these two periods is 39·5; that of the second is 42·3. The mean aggregate rainfall of the first is 8·4 inches; that of the second, as already mentioned, is 6·7 inches. Now if cold were the only cause of catarrh, or the only meteorological condition affecting its prevalence, the three coldest months should include a larger number of attacks than the three driest months; but the reverse is the case, the three driest months including the larger number, in the proportion of 31 to 24. It would therefore appear as if a deficient rainfall were really an effective agency in promoting the prevalence of catarrh.

Pursuing the comparison of the three coldest months with the three months of least rainfall, it may be well to inquire how the two periods are related in respect of atmospheric humidity. The mean humidity of December, January, and February I find to be 87·4 (saturation = 100); the mean humidity of February, March, and April is 83·3. The comparative frequency of catarrh, therefore, in the three last-named months may be either due to deficient rainfall, or to atmospheric dryness, or to both combined. The three driest months of the year in respect of atmospheric humidity are May, June, and July; but the higher temperature of these months overrides and obscures all other influences.

There is still another comparison that may be made between the three coldest months and the three months of least rainfall. I refer to the relative prevalence of north-easterly and south-westerly winds. Calling all winds north-easterly which blow from points between north and east inclusive, and all south-westerly which blow from points between south and west inclusive, I find that while in the three coldest months south-westerly winds prevail over north-easterly in the proportion of 32 to 24, in the three months of least rainfall north-easterly winds prevail over south-westerly in the proportion of 29 to 28. Here, then, is a third point of difference between the two periods now under comparison, and one which may claim, equally with the difference of rainfall and the difference of atmospheric humidity, a share of influence in bringing about the difference observed in the prevalence of catarrh—a difference which we have seen to be in a direction opposite to that which temperature alone should occasion.

To sum up the results which seem deducible from my figures, I would begin by throwing out two suggestions of a negative character, both in opposition to current opinions. First, if "getting wet" be an influential cause of catarrh, it is difficult to see why catarrhs should be most frequent in the months when rainfall is least. Secondly, if cooling of the body after free perspiration be a fruitful cause, it is strange that the fewest catarrhs should occur in the warmest months.

The positive conclusions at which I arrive are the following:—

First, catarrh is promoted by the cold of winter, and is greatly checked by the heat of summer.

Secondly, it is promoted by one or more of three causes which occur together—namely, deficient rainfall, dryness of air, and north-easterly wind.

But I need hardly point out how unphilosophical it would be to infer from these results that either the cold, or the dryness, or the north-easterly wind is the essential cause of catarrh. Plague, cholera, and yellow fever are all in a marked degree under the influence of atmospheric conditions, notably of temperature; yet no one believes that either of these diseases is the direct product of heat, or hesitates to assign it a place amongst the zymotic group.

I apply the same principles to catarrh. I believe it to have its origin in a specific organism, the germs of which float in the air. This organism, like other organisms, is dependent for its activity and propagation upon atmospheric conditions. Unlike many, it thrives in cold and languishes in heat. Dry north-easterly winds bring to it life and vigour; while south-westerly winds depress its energies, and the rains which accompany them wash it away. Drawn into the air passages with the breath, it lodges in the mucous membrane, where probably it multiplies and spreads, giving rise to the local symptoms of a cold. There may be nothing more than this, or the organism, penetrating the tissues, may find its way into the blood, and, by its presence in the circulating fluid (where again it multiplies), may set up a state of fever.

To conclude, if the subject of my discourse seem comparatively trivial, I may be allowed to plead that in science nothing is trivial; and that as no error can fail to be hurtful, so no truth can fail to be helpful, and that in a degree not to be measured by the intrinsic importance of the subject to which the truth or the error immediately refers.

